# Additional evaluations show that specific *BWA‐aln* settings still outperform *BWA‐mem* for ancient DNA data alignment

**DOI:** 10.1002/ece3.8297

**Published:** 2021-12-17

**Authors:** Adrien Oliva, Raymond Tobler, Bastien Llamas, Yassine Souilmi

**Affiliations:** ^1^ Australian Centre for Ancient DNA School of Biological Sciences Faculty of Sciences The University of Adelaide Adelaide South Australia Australia; ^2^ The Environment Institute Faculty of Sciences The University of Adelaide Adelaide South Australia Australia; ^3^ National Centre for Indigenous Genomics Australian National University Canberra Australian Capital Territory Australia

## Abstract

Xu et al. (2021) recently recommended a new parameterization of *BWA‐mem* as a superior alternative to the widely‐used *BWA‐aln* algorithm to map ancient DNA sequencing data. Here, we compare the *BWA‐mem* parameterization recommended by Xu et al. with the best‐performing alignment methods determined in the recent benchmarks of Oliva and colleagues (2021), demonstrating that *BWA‐aln* is still the gold‐standard for ancient DNA read alignment .

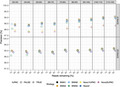

## 1 INTRODUCTION

Xu et al. (2021) recently suggested a new parameterization of *BWA*‐*mem* (Li, 2013) as an alternative to the current standard *BWA*‐*aln* (Li & Durbin, 2009) to align ancient DNA sequencing data. The authors tested several combinations of the ‐*k* and ‐*r* parameters to optimize *BWA*‐*mem's* performance with degraded and contaminated ancient DNA samples. They report that using *BWA*‐*mem* with ‐*k* 19 ‐*r* 2.5 parameters results in a mapping efficiency comparable to *BWA*‐*aln* with ‐*I* 1024 ‐*n* 0.03 (i.e., a derivation of the standard parameters used in ancient DNA studies; (Schubert et al., 2012)), while achieving significantly faster run times.

We recently performed a systematic benchmark of four mapping software (i.e., *BWA*‐*aln*, *BWA*‐*mem*, *NovoAlign* (http://www.novocraft.com/products/novoalign), and *Bowtie2* (Langmead & Salzberg, 2012)) for ancient DNA sequencing data and quantified their precision, accuracy, specificity, and impact on reference bias (Oliva et al., 2021). Notably, while multiple parameterizations were tested for *BWA*‐*aln*, *NovoAlign*, and *Bowtie2*, we only tested *BWA*‐*mem* with default parameters.

Here, we use the alignment performance metrics from Oliva et al. to directly compare the recommended *BWA*‐*mem* parameterization reported in Xu et al. with the best performing alignment methods determined in the Oliva et al. benchmarks, and we make recommendations based on the results.

## 2 METHODS

We investigated the alignment performance of the parameterization recommended by Xu et al. (2021), that is, ‐*k* 19 and ‐*r* 2.5 (hereafter called BWA9) against several of the best performing strategies identified in Oliva et al. (namely, BWA1, BWA2, BWA8, Novo1IUPAC, Novo2IUPAC, and Novo2, see Table 1 for parameter settings).

**TABLE 1:** Different alignment parameterizations tested. To simplify comparisons with the results reported in Oliva et al. (2021), we reuse the alignment strategy labels from that study

Strategy	Software	Parameterization
BWA1	*BWA‐aln*	‐l 1024 ‐n 0.01 ‐o 2
BWA2	*BWA‐aln*	‐l 1024
BWA8	*BWA‐mem*	default
BWA9	*BWA‐mem*	‐k 19 ‐r 2.5
Novo1IUPAC	*NovoAlign*	‐k
Novo2(IUPAC)^a^	*NovoAlign*	default

^a^ Used with and without the IUPAC reference (Novo2 and NovoIUPAC).

Following the analytical framework of Oliva et al., our benchmark is based on simulated reads (including fragmentation, damage, and sequencing errors typical for ancient DNA samples; see (Oliva et al., 2021)) that were generated for each of the following three samples from the 1000 Genome Project dataset (1000 Genomes Project Consortium et al., 2015), each coming from a distinct population, and were aligned to reference genome GRCh37:

- Individual *HG00119* from the British in England and Scotland population; GBR; labeled Europe in this study.
- Individual *NA19471* from the Luhya population in Webuye, Kenya; LWK; labeled Africa in this study.
- Individual *HG00513* from the Han Chinese population in Beijing, China; CHB; labeled East Asia in this study.

In addition to quantifying read alignment precision (i.e., the proportion of correctly aligned reads relative to all aligned reads) and proportion of aligned reads (i.e., the fraction of aligned reads relative to the total number of simulated reads) for each strategy, we tested the specificity (i.e., the fraction of unmapped reads) of these strategies for two sets of potential contaminants—that is, bacterial and dog reads—that were also used in Oliva et al. (2021).

## 3 RESULTS

**FIGURE 1:**
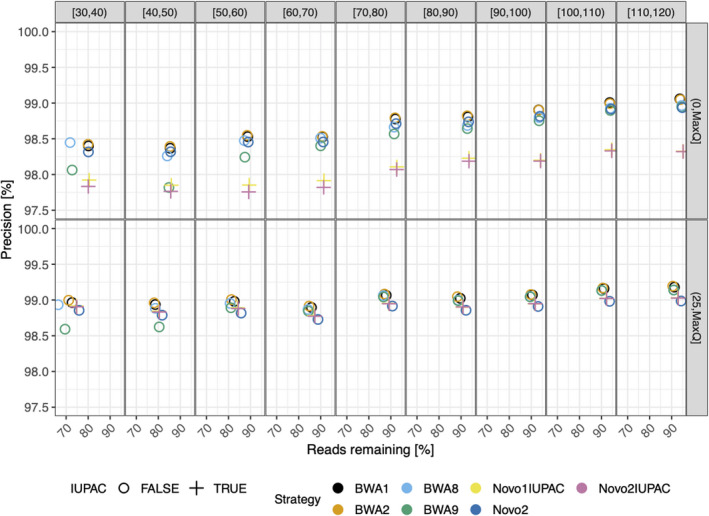
Alignment precision relative to read length and mapping quality for the simulated East Asian sample. Results are shown for seven parameterizations of four different alignment software, including an IUPAC reference‐based alignment for a subset of the *NovoAlign* parameterizations (see key). BWA9 is the *BWA*‐*mem* strategy recommended by Xu et al. (2021), with parameter details for the other strategies provided in Table 1. The panels in each row show results after applying the specific mapping quality filter, which results in the removal of all reads below the required mapping quality. Results were similar for the simulated European and African samples and are shown in Figures A1 and A2, respectively

**FIGURE A1:**
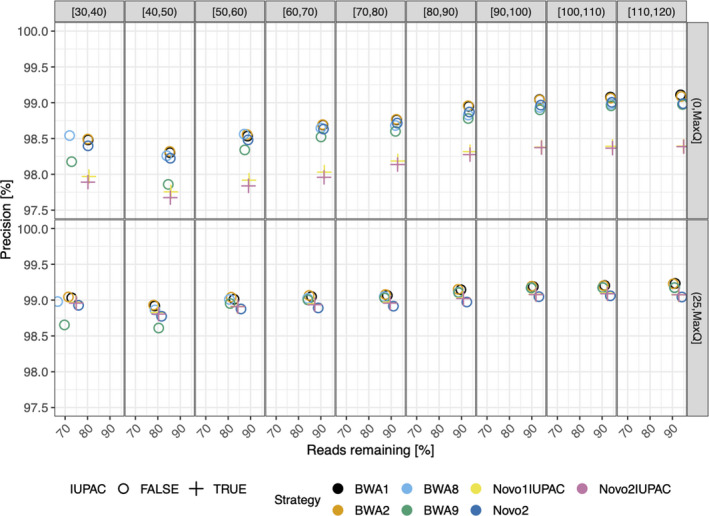
Alignment precision relative to read length and mapping quality for the simulated European sample. See Figure 1

**FIGURE A2:**
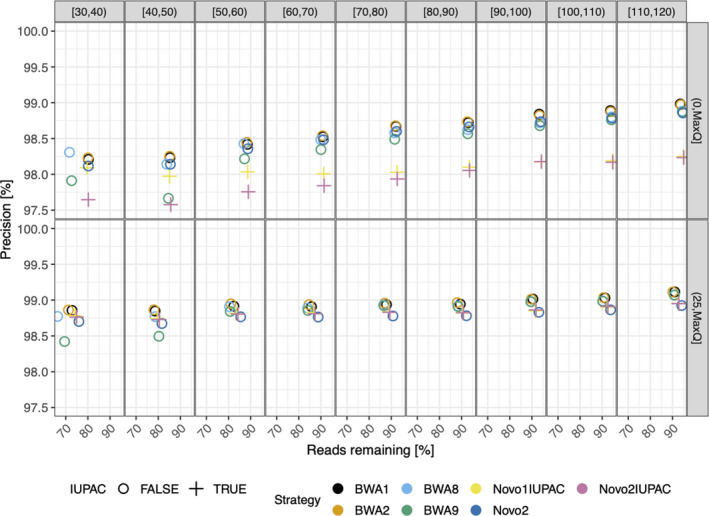
Alignment precision relative to read length and mapping quality for the simulated African sample. See Figure 1

BWA9 had a slight improvement in the proportion of total reads aligned relative to *BWA*‐*mem* using default settings (BWA8), but this came at the cost of consistently lower precision (Figure 1, Figures A1 and A2). These precision differences are particularly accentuated for reads between 30 and 60bp, the range of read lengths that is typical of ancient DNA. As demonstrated here and in more detail in our recent alignment software benchmark (Oliva et al., 2021), *BWA*‐*aln* (BWA1 and BWA2) is the most precise alignment method among the tested strategies, having moderately higher precision relative to *BWA*‐*mem* for shorter reads while mapping a much higher percentage of reads overall (Oliva et al., 2021; van der Valk et al., 2021).

**FIGURE 2:**
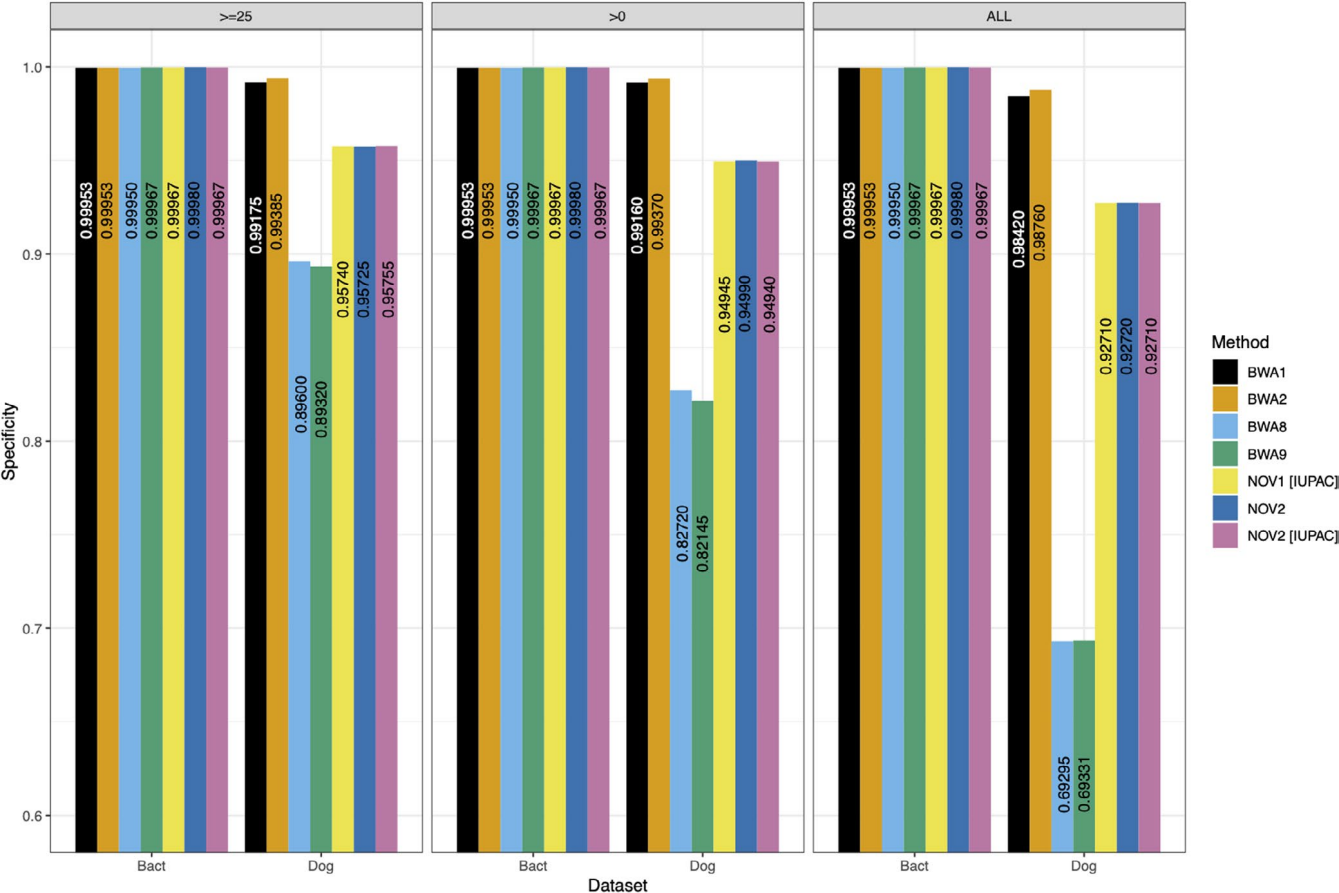
Specificity of all tested alignment methods. Bacterial and dog reads were aligned to the GRCh37 reference using the seven tested parameterizations of four different alignment software, including an IUPAC reference‐based alignment for a subset of the *NovoAlign* parameterizations (see key). The specificity corresponds to the number of unmapped reads, with higher values being better

When comparing specificity against potential contaminants, BWA9 has a near‐identical specificity to the default *BWA*‐*mem* parameterization (BWA8) for dog reads, and slightly poorer specificity when testing with bacterial reads, but both parameterizations perform considerably worse than the tested *NovoAlign* (Novo1IUPAC, Novo2IUPAC, and Novo2) and *BWA*‐*aln* (BWA1 and BWA2) strategies for dog reads (Figure 2).

**FIGURE 3:**
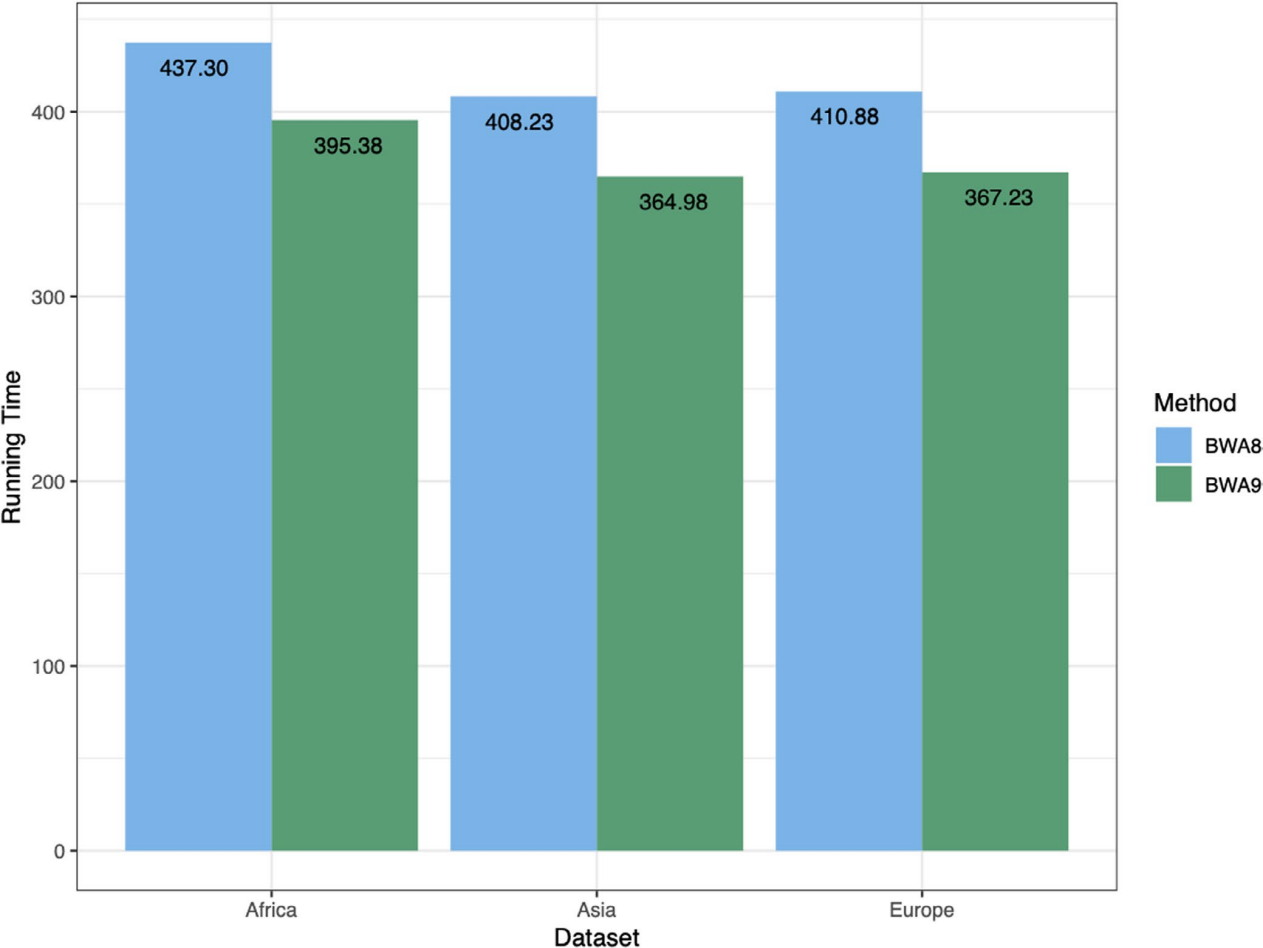
Execution time for each of the *BWA*‐*mem* strategies. The execution time (walltime) in seconds of BWA8 (default parameters) and BWA9 (Xu et al., 2021 parameterization; ‐k 19 ‐r 2.5) based on 1.5 million simulated reads

Finally, comparing running times of the two *BWA*‐*mem* parameterizations for each of the three simulated human datasets showed that BWA9 is slightly quicker than BWA8 (Figure 3), confirming the results of ref. (Xu et al., 2021).

## 4 CONCLUSION

Xu et al. (2021) report that *BWA*‐*mem* produces alignment results that are comparable to a derivation of *BWA‐aln* widely used in the ancient DNA field. Consequently, they recommend the use of a specific non‐default *BWA*‐*mem* parameterization for ancient DNA studies because of its superior runtime relative to *BWA*‐*aln*. However, we find that this parameterization actually decreases alignment precision relative to *BWA*‐*mem* using default settings for sequencing reads shorter than 70 bases, which are particularly abundant in ancient DNA samples. Moreover, *BWA*‐*mem* is consistently outperformed by *BWA*‐*aln* under the tested parameterizations for both precision and the proportion of reads mapped, and also had greatly improved specificity when the DNA contamination came from a phylogenetically related organism (i.e., a dog in the present study). Crucially, Oliva et al. have demonstrated that improvements in these alignment metrics are also complemented by a reduction in reference genome bias—an alignment‐related bias resulting from the preferential mapping of alleles on the reference genome (relative to alternate alleles) that can inflate false positives and is particularly problematic for ancient DNA studies.

**FIGURE A3:**
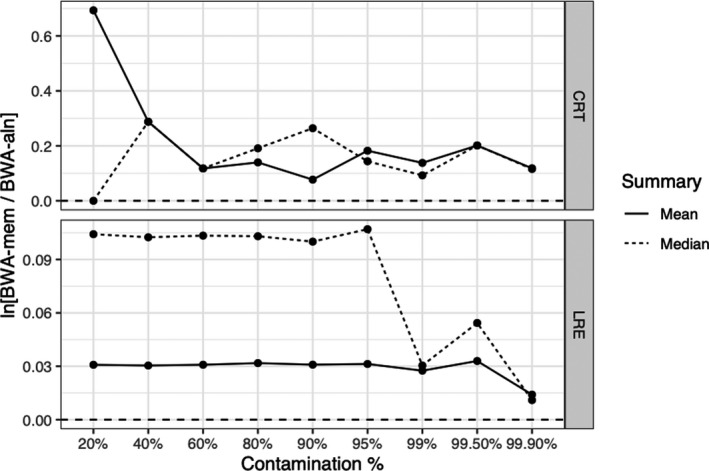
Summary of alignment performance of *BWA*‐*mem* relative to *BWA*‐*aln* across increasing levels of contamination from results reported in Xu et al. supplementary table 4. Xu et al. (2021) summarise alignment results using two statistics: (1) the contamination rate after treatment (CRT; top panel), which measures the proportion of aligned contaminants relative to all contaminant reads, and (2) the loss rate of endogenous DNA (LRE; bottom panel), which records the proportion of unmapped endogenous reads relative to all endogenous reads. Notably, the reported mean and median values for these both metrics are consistently higher for *BWA*‐*mem* relative to *BWA*‐*aln* – as shown by the natural logarithm of the ratio of *BWA*‐*mem* to *BWA*‐*aln* being consistently above 0 (dashed line) for both metrics – indicating that *BWA*‐*mem* tends to map more contaminant reads and less endogenous reads than *BWA*‐*aln* across all tested contamination levels, whereby *BWA*‐*mem* has poorer overall performance

The recommendations of Xu et al. (2021) are based on the lack of statistical differences between the alignment performance of *BWA*‐*mem* and *BWA*‐*aln* evaluated using a repeated‐measures ANOVA approach, whereby they recommend *BWA*‐*mem* because of its faster execution. However, when re‐examining the alignment performance results reported in supplementary table 4 of Xu et al. (2021) we find that *BWA*‐*aln* maintains a small but consistent advantage over *BWA*‐*mem* across different levels of contamination for both tested alignment metrics (see Figure A3)—a result that is consistent with the findings in the present study using complementary metrics. Indeed, the lack of statistical support for the difference between the two alignment algorithms most likely results from the effect size being small relative to the variance observed across the tested replicates used in the Xu et al. study (see Figure 2 in Xu et al., 2021), leading to insufficient power to detect these differences.

Taken together, our results indicate that the *BWA*‐*aln* strategies tested here provide a small but consistent improvement over the *BWA*‐*mem* parameterization recommended by Xu et al. (2021) for simulated aDNA read datasets when evaluated using the complimentary sets of metrics employed in Xu et al. (2021) and the present study. Importantly, while the differences between the two alignment methods are relatively small (on the order of 0.1–0.5%; see Figure 1), they are sufficient to inflate reference bias in downstream analyses that can negatively impact inferences (Oliva et al., 2021).

Accordingly, despite having improved run times, we do not recommend that *BWA*‐*mem* be prioritized over *BWA*‐*aln* for research using short reads—such as ancient DNA, cell‐free DNA, and forensic research fields. If run time is an issue for researchers, we recommend the use of *NovoAlign* using the free default parameterization, so long as an appropriate IUPAC reference can be generated. Readers interested in a more detailed discussion of these issues are directed to refs. (Oliva et al., 2021; Poullet & Orlando, 2020; Schubert et al., 2012; van der Valk et al., 2021) for recent benchmarks of different alignment strategies using short reads.

## CONFLICT OF INTEREST

The authors declare no conflict of interest.

## AUTHOR CONTRIBUTIONS

**Adrien Oliva:** Conceptualization (equal); Data curation (equal); Formal analysis (equal); Investigation (equal); Methodology (equal); Writing‐original draft (equal). **Raymond Tobler:** Supervision (equal); Writing‐review & editing (equal). **Bastien Llamas:** Supervision (equal); Writing‐review & editing (equal). **Yassine Souilmi:** Conceptualization (equal); Supervision (equal); Writing‐review & editing (equal).

## Data Availability

The scripts used to create the used datasets in this study are available in the github repository at: https://github.com/AdrienOliva/Benchmark‐aDNA‐Mapping.
